# Mr. Simmons, on Arsenic in Cancer

**Published:** 1801-03

**Authors:** W. Simmons

**Affiliations:** Manchester


### *To the Editors of the Medical and Physical Journal.*

Gentlemen,

TWO years have elapsed since I published a short account of a case of Cancer then under treatment, in which the solution of white arsenic appeared to have acted as an *anodyne*. That case, as might be expected, proved fatal, but the remedy did not disappoint my prediction. I have now a second patient under a course of the same remedy, where my expectations respecting it have been fully realized. The subject is a woman, in the fifty-third year of her age, whose left breast, adherent to the pectoral muscle, and in a cancerous state, together with a large cluster of indurated glands in the axilla, I extirpated in March last. The integuments covering a part of the breast had become diseased, so that union by the first intention could not be accomplished. Nevertheless, the parts healed completely, and for several weeks she continued apparently well. The return of the disease was then manifested by a small vesication, which terminated in a painful ulcer, about the size of her finger nail, in the middle of the cicatrix formed by the operation, and by painful indurations surrounding it, and in the course of the absorbents leading to the axilla on the same side. With the disease slowly advancing, she suffered for several weeks longer before she applied again for help, when she complained of pain all over the ulcerated surface, and particularly in the knots, especially when pressed.

She had been forewarned of the probable recurrence of the disease, owing to the too long delay of the operation, which was performed with proper regard to every circumstance of candour and caution.

On the 14th of July, when I first put her upon the use of the mineral solution, the ulceration was extending itself rapidly, and the pain all over the surface of the ulcer was constant and very severe, as well as in the indurated parts. She had not taken it more than nine days, in doses of twelve drops, three times a day, before she thought the pain sensibly decreased. To the ulcer itself, the ung. ceræ cum opio was directed; but as, after a sufficient trial, she thought the pain aggravated by it, the infus. cicutæ was substituted, as in the former case.

It is now upwards of six months since she began the use of the arsenical solution, which has been taken in the above doses with great regularity till within a few days. This suspension of it has been recommended on account of a pain in the head, and of an uneasiness in her stomach, both of which she ascribes

 to to a monthly conftitutional change now not outwardly marked; and as they had nearly fubfided this morning, I am inclined to favour her opinion of the caufe. Notwithftanding the ulcerated parts had fo long ceafed to be painful, the cicatrization was not much advanced till fhe came into the Infirmary, where fhe was a fecond time admitted an in-patient, under the cancer-plan, on the 12th of January, 1801.

There is now very little ulceration, and the fkinning is ftill progreffively advancing; the pain too, which is not conftant, is confined to the knots, fome of which are inflamed, and going to exfoliate. In Graham's cafe it will be recollected, that this was the fource of pain, after fhe had for fome time taken the arfenical folution.

I have purpofely compreffed the recital of this cafe, and confined myfelf to the leading points of it; but it muft be again obferved, that the ulceration was confiderable, and very painful.

The drops were at firft taken in a little water, but, on account of their feeming to difagree with her ftomach, it was changed for pepper-mint-water, which at that time had the defired effect. Latterly, fhe has been allowed four ounces of red wine daily; in other refpects, fhe has taken only the common diet of the houfe.

No opium has been adminiftered, though, when the functions of the ftomach were difturbed, I thought it indicated, left its exhibition fhould throw any ambiguity over the refult; however, I am difpofed to think that it may become neceffary, to enable her to bear the mineral folution in proper dofes. Henceforward, I fhall not fcruple to employ it, fhould the fymptoms require its ufe, in conjunction with the drops. And fhould chronic general inflammation, which has been faid to attend the long continued exhibition of arfenic, be excited, but which I have not feen, the digitalis offers a refource admirably calculated to reprefs it.

Thefe are the feveral means by which, in the prefent inftance, I propofe to combat this formidable difeafe.

Partial fuccefs has already attended my endeavours, and I expect not to accomplifh a cure. Among the multiplicity of noftrums for cancer, vaunted by empirics, arfenic is probably the active ingredient; it was formerly much employed, but had, I believe, been difcarded from regular practice. My own experience proves it to be fafe, when cautioufly adminiftered; for I have given it, in another difeafe, to a child of fifteen months old, and with fuccefs.

Your judicious readers will form their own conclufions from my *two* cafes: My own are;—

1. That

1. That arsenic does diminifh pain, and promote the healing of an ulcerated cancer;

2. That it may be fafely adminiftered, in proper dofes, for a great length of time, without endangering life, or even exciting any of thofe alarming fymptoms which have been faid to attend its continued exhibition.

This brief notice you will oblige me by laying before the public, from whom, confiftently with my own feelings for the fufferings of thofe who may be afflicted with Cancer, I could not any longer withhold it.

I have the honour to be, &c.

